# Q-SEA – a tool for quality assessment of ethics analyses conducted as part of health technology assessments

**DOI:** 10.3205/hta000128

**Published:** 2017-03-15

**Authors:** Anna Mae Scott, Björn Hofmann, Iñaki Gutiérrez-Ibarluzea, Kristin Bakke Lysdahl, Lars Sandman, Yvonne Bombard

**Affiliations:** 1 Centre for Research in Evidence-Based Practice (CREBP), Bond University, Gold Coast, Queensland, Australia; 2 Institute for the Health Sciences, Norwegian University for Science and Technology, Gjøvik, Norway; 3 Centre of Medical Ethics, University of Oslo, Oslo, Norway; 4 Dept. of Biochemistry, Nursing University School Vitoria-Gasteiz, Vitoria-Gasteiz, Basque Country, Spain; 5 OSTEBA, Basque Office for HTA, Research and Innovation Directorate, Department for Health, Vitoria-Gasteiz, Basque Country, Spain; 6 Centre for Medical Ethics, University of Oslo, Oslo, Norway; 7 National Centre for Priority Setting in Health Care, Linköping University, Linköping, Sweden; 8 University of Borås, Borås, Sweden; 9 Li Ka Shing Knowledge Institute of St. Michael's Hospital, Toronto, Canada; 10 Institute of Health Policy, Management and Evaluation, University of Toronto, Toronto, Canada

**Keywords:** health technology assessment, systematic reviews, ethics analysis, quality assessment, methodology

## Abstract

**Introduction:** Assessment of ethics issues is an important part of health technology assessments (HTA). However, in terms of existence of quality assessment tools, ethics for HTA is methodologically underdeveloped in comparison to other areas of HTA, such as clinical or cost effectiveness.

**Objective:** To methodologically advance ethics for HTA by: (1) proposing and elaborating Q-SEA, the first instrument for quality assessment of ethics analyses, and (2) applying Q-SEA to a sample systematic review of ethics for HTA, in order to illustrate and facilitate its use.

**Methods:** To develop a list of items for the Q-SEA instrument, we systematically reviewed the literature on methodology in ethics for HTA, reviewed HTA organizations’ websites, and solicited views from 32 experts in the field of ethics for HTA at two 2-day workshops. We subsequently refined Q-SEA through its application to an ethics analysis conducted for HTA.

**Results:** Q-SEA instrument consists of two domains – the process domain and the output domain. The process domain consists of 5 elements: research question, literature search, inclusion/exclusion criteria, perspective, and ethics framework. The output domain consists of 5 elements: completeness, bias, implications, conceptual clarification, and conflicting values.

**Conclusion:** Q-SEA is the first instrument for quality assessment of ethics analyses in HTA. Further refinements to the instrument to enhance its usability continue.

## Introduction

Health Technology Assessment (HTA) focuses on the “medical, economic, social, and ethical implications of the development, diffusion, and use of health technologies”, such as drugs, medical devices, screening tests and medical procedures [1], [2], [3]. However, while ethics is a recognised dimension of HTA, it is frequently regarded as methodologically less well-developed than other areas of HTA, such as clinical or economic effectiveness [4], [5], [6], [7], [8], [9].

This is not entirely correct, as numerous methodological advances *have* occurred in this space over the last decade, including: development of methods for integrating ethics issues into health technology assessments, search strategies for identifying literature on ethics issues, and advances in methodology for identifying *when* an assessment of ethics issues ought to be conducted as part of an HTA. Nevertheless, one area of ethics for HTA that indeed is considerably underdeveloped in comparison to other areas of HTA is quality assessment of ethics analyses in HTA [10], [11]. 

A wide spectrum of ‘ethics analyses’ can be conducted for HTA purposes [12]. The most basic type of ethics analysis consists of presenting several arguments for or against a particular health technology, without claims to comprehensiveness – often found in early commentaries or as added information in articles on new technologies, such as uterus transplantation [13], [14]. A more sophisticated analysis presents a systematic review of the relevant arguments for and against the technology, leaving to the reader to decide whether overall, the arguments support or oppose the technology [15]. Still more sophisticated analyses would additionally introduce conceptual clarifications, and relate arguments to more basic norms and values – reaching a normative conclusion about the ethical acceptability of the technology, and potentially also analysing whether this conclusion varies according to the adopted perspective – e.g. the patient’s, the healthcare provider’s, the health system’s, etc. [12]. Examples of these types of ethics analysis can be found in the literature [16], [17]. 

However, different quality criteria apply to different types of analyses – much like different quality criteria apply to different types of epidemiological studies. Thus, it is important to be clear about the target of the proposed quality assessment instrument. Our focus is on proposing and demonstrating Q-SEA, the first instrument for quality assessing ethics analyses at the last two levels. This article therefore first elaborates Q-SEA, and second, applies it to a sample systematic review, in order to illustrate and facilitate its use. 

## Methods

To generate the instrument, we adopted a rigorous, three-pronged process, which we have reported previously [10]. Briefly, first, we identified a recent systematic review of guidelines for integrating ethics issues into health technology assessments [18], and reviewed in full all of the included guidelines, searching for criteria for assessing the quality of ethics analyses. Second, we updated its search strategy, identifying 420 further documents, which on application of pre-established inclusion and exclusion criteria, identified 42 documents for full-text review, and 6 documents for inclusion. Although all 6 recognised the importance of quality in conducting ethics analyses, no formal instrument for assessing such quality was proposed. Finally, we also convened two 2-day workshops of experts in methodology in ethics for HTA, which focused on the issue of quality assessment in ethics for HTA. The workshops yielded a preliminary list of elements for Q-SEA; the list subsequently underwent multiple rounds of refinements over email discussions – indeed, undergoing further refinements from the list of items initially proposed [10]. This, finally, resulted in the Q-SEA instrument presented below. 

## Results

### Q-SEA: An instrument for the quality assessment of ethics analyses

Q-SEA – Quality Standards for Ethics Analyses – instrument is proposed for assessing the quality of ethics analyses conducted for HTA purposes. The instrument consists of elements categorised into two domains: the process domain and the output domain (Table 1 (Tab. 1)). 

#### The Process Domain

The ‘process’ domain focuses on assessing the quality of the elements that constitute the process of arriving at the ethics analysis. The elements in this domain include: research question, literature search, inclusion and exclusion criteria, perspective, and ethics framework. 

##### i) Research Question

Although the issue of defining a research question in systematic reviews of ethics issues has been a matter of some controversy [19], approaches similar to systematic reviews of clinical evidence have more recently become generally accepted. For example, McCullough et al. [17] recommended that a normative systematic review of clinical ethics literature should address a clinical question in the PICO format – for example, “In [P] patients with mental disorders, [I] is use of concealed medications in food/drink, [C] rather than prescribing medications in the usual way or forcibly administering them, [O] ethically justifiable?” Similarly, in a systematic review of descriptive ethics, one could ask: “What are the ethics issues relevant for the assessment, deliberation, decision making, implementation, and use of non-invasive prenatal testing in antenatal care?” Both normative and descriptive ethics analyses lend themselves to clearly stated research questions; we therefore remain agnostic on whether the research question needs to be in the PICO format. However, it is worth noting that formulation of research questions in PICO format enables ready translation into search and information retrieval strategies commonly used in HTA. 

##### ii) Literature search

The systematic review of ethics issues in HTA – much like systematic reviews of clinical evidence – require a comprehensive search of sources. The comprehensiveness of a search depends both on how the search strategy is designed and on the selection of sources of information [20]. A comprehensive search of ethics issues around a health technology makes use of a variety of sources, including biomedical databases (Medline, EMBASE etc.), nursing databases (e.g. CINAHL), systematic reviews databases (e.g. the Cochrane library, CRD-HTA, EuroScan database), Social Sciences and Psychology databases (e.g. PsycInfo), and databases with philosophical and ethical content (e.g. PhilPapers, BELIT, SIBIL, ETHICSWEB). Both international and national sources should be searched, due to the context sensitivity of ethical aspects. In addition to the electronic databases, it is recommended to hand search non-indexed journals (e.g. Clinical Ethics) and anthologies. Searching the reference lists of already included publications or using a ‘related articles’ feature (snowballing or hand-searching) can also increase the comprehensiveness of the search. As in all reviews, the time period and language restrictions (if any) of the searches should be clearly stated and justified if potential for bias exists. 

##### iii) Inclusion and exclusion criteria

The requirements for inclusion and exclusion criteria in systematic reviews of ethics issues are similar to systematic reviews of other types of evidence. Predefined criteria should be clearly described and aligned with the research question. The inclusion and exclusion criteria may be less strict regarding type of publication than systematic reviews of clinical effectiveness, as ethical arguments may very well be found in commentaries or editorials, for example. The most important criterion is that the publication provides relevant arguments and assessment that can illuminate moral aspects of the technology [21]. Hence, a relevant exclusion criterion is publications where ethics issues are considered only briefly and tangentially [22]. Lack of thoroughness of ethical arguments may also be an exclusion criteria [23]. The setting of the intervention can also be a relevant criterion for an ethics analysis – for example including only publication focusing on hospital-based health professionals in developed countries [24].

##### iv) Perspective

Generally, an ethics analysis should be conducted from an impartial perspective, analysing how the health technology affects the relevant values and norms for different stakeholders. However, there are cases where it is appropriate for an ethics analysis to adopt a specific perspective – for example, a healthcare system perspective, patient perspective, societal perspective, etc. For example, if a health technology under assessment has the potential to intrude on the privacy of patients, an analysis looking at privacy issues from the perspective of patients might be an acceptable. However, conducting an ethics analysis from a specific stakeholder perspective does not imply simply asking about stakeholder views; the outcome of the ethics analysis might be at odds with stakeholder views. In that sense, an ethics analysis from a specific perspective is still impartial. As in the case of economic or qualitative analyses, where an ethics analysis *is* conducted from a specific stakeholder perspective, this should be made clear to the reader.

##### v) Ethics framework

A wide range of frameworks (also sometimes referred to as approaches, methods, positions) may be applied in ethics analyses conducted for HTA. These include: the Socratic approach, principlism, casuistry, coherence analysis, participatory HTA approach (iHTA), and others [25]. What is an appropriate framework to use will vary by context and the technology under assessment, but tools for choosing an ethics framework for analyses of ethics issues for some types of health technologies (e.g. complex health interventions) already exist [26] and can be utilised. Other factors relevant to the choice of framework may include: local preferences, expertise of those conducting the analysis, time and financial constraints, and so on. Nevertheless, whatever framework is selected, a well conducted ethics analysis ought to clearly identify the applied framework(s). Clarity about the choice of framework is important as a certain argument about a health technology may be understood differently if it appears in a utilitarian context compared to in a deontological context. Explicit identification of the adopted framework may therefore also help to avoid “interpretation bias”. 

#### The Output Domain

The ‘output’ domain focuses on assessing the quality of the elements that constitute the output – that is, the ethics analysis that is the result of the process. The elements included in the output domain are: completeness, bias, implications, conceptual definitions, and conflicting values. 

##### i) Completeness

Systematic reviews of ethics issues pose a challenge with respect to completeness – how to know when all relevant arguments and issues have been identified. To ensure completeness of ethics analyses, some authors have used qualitative research approaches [27], including thematic analysis, meta-ethnography and content analysis [28], [29]. Ring et al. report that meta-ethnography, meta-study, meta-summary, and thematic synthesis were the most used methods and could be a good starting point when performing a review of reasons [30]. Thus, a wide range of methods can be appropriate here, and the particular methodology adopted will vary by factors such as the specific question to be addressed, resources, and expertise of the systematic reviewers. However, it is also crucial for an adequately performed ethics analysis to reflect on whether additional – unidentified in the literature search – relevant ethics issues are raised by the technology. Identification of gaps in the state of knowledge is especially crucial for new and emerging technologies, but also arises for well-established technologies. If it is unlikely that gaps in the literature around ethics issues pertaining to specific technology exist, this should be explicitly indicated, with reasons for this determination. 

##### ii) Bias

Like other types of analyses, ethics analyses may be subject to bias. Therefore, biases should be explicitly identified and discussed in an ethics analysis. A series of potential biases can arise whilst conducting an ethics analysis for HTA purposes. “Interpretation bias” has already been mentioned. Bias in the selection of end-point (“end-point-selection bias”), “perspective bias”, “search bias”, and bias in the presentation of arguments, are but a few examples of potential biases in ethics analysis. Which biases are relevant for an assessment of ethics issues around a specific health technology in a given health care and HTA context may vary greatly. However, crucial here is that the existence of biases (or potential biases) is acknowledged, the types of biases identified, and discussed in an ethics analysis. 

##### iii) Implications

Implications of the ethics analysis need to be clearly and explicitly presented, as well as differentiated by stakeholder group (e.g. patients, healthcare professionals, industry, etc.). There are differences here with respect to descriptive systematic reviews and normative ones. Although descriptive systematic reviews aim to identify the issues that are relevant for decision-making, and do not aim to make recommendations, they are still able to identify the implications of the various ethics issues identified, without making prescriptive recommendations. Conversely, for normative ethics analyses, it is crucial that the arguments for specific recommendations are clearly identified and justified. Similar considerations apply to differentiating implications by stakeholder groups. Analyses that only address the ethical implications for one stakeholder group are of poorer quality than analyses that address implications for multiple stakeholders. 

##### iv) Conceptual clarification

Ethical concepts are generally “thick concepts” – they have both a descriptive content and a strong evaluative component. Thus, their ambiguity and complexity requires clarification. To some extent, the ethics framework chosen to conduct the analysis may provide clarifications of ethical concepts, such as beneficence, dignity, etc. However, the degree (if any) of conceptual clarification provided by a framework might not be sufficient for use in the specific analysis of ethics issues, and even where the conceptual clarifications are provided, it is worth noting that the same concepts may be differently understood by different ethical frameworks. Moreover, an important part of conceptual clarification is to assess whether concepts used have been applied consistently through the ethics analysis. Methodology for this type of conceptual clarification exists – e.g. Brülde’s method of conditions of adequacy for conceptual clarification. Brulde’s list includes the following conditions: the ordinary language condition – relating it to ordinary language use; the value condition – whether the value-ladenness of the concept is explained; the coherence condition – whether the concept is applied consistently; the precision condition – whether the concept is precise enough; the reliability condition – whether the concept is used in a transparent way; and the simplicity condition – whether the concept is simple enough [31]. 

##### v) Conflicting values

Values and norms involved in ethical arguments regarding a specific health technology can be implicit or explicit. For example, in the assessment of a new type of assisted reproductive therapy such as mitochondrial donation, conflicts may arise between the values of reproductive choice and caution in the face of uncertainty. These value conflicts may be made explicit or left implicit in an ethics analysis. However, where they are left implicit, this may lead to an unwarranted interpretation of the results of the ethics analysis, biases, and/or in misleading conclusions. High quality ethics analysis therefore ought to explicitly identify the conflicts of values; whether it is appropriate to both identify *and balance* these conflicts, will depend on whether the systematic review of ethics issues is descriptive or normative. 

#### Overall quality assessment 

In line with the approach in the *Cochrane Handbook*, we recommend avoiding the scale-based approach – that is, one in which the elements of the quality assessment tool are scored individually, and combined in an overall score [32]. Instead, we recommend that both the process domain and the output domain – together with their constitutive elements – be critically evaluated, and results presented narratively. 

### Application of the Q-SEA instrument

In order to facilitate the use of the Q-SEA instrument, we present here its application to a systematic review of ethics issues in autologous stem cell transplantation (ASTC). This systematic review of ethics issues was conducted as a result of identification of ethics issues surrounding ASTC during a systematic review of the effectiveness of ASTC by the German Institute for Quality and Efficiency in Health Care (IQWiG) [23]. 

#### The Process Domain

##### i) Research Question

An ethics analysis should have a clearly stated research question. Droste et al. clearly state that their focus is on “ethical issues related to ASTC [autologous stem cell transplantation] in locally advanced and metastatic breast cancer patients” [23]. Although the research question is not formulated in the PICO format, three of the components of the PICO format are readily identifiable – the population are the patients with locally advanced and metastatic breast cancer; the intervention is ASTC; and the outcomes are ‘ethical issues’. 

##### ii) Literature search

The literature search is very comprehensive, aiming for high sensitivity of the searches. Searches included both domestic (German) and international sources. Twenty-seven databases were searched, in the following subjects: biomedicine, nursing, psychology, social sciences, health economics, ethics, monographs, and publisher databases. Additionally, snowballing and citation tracking was carried out (without further description how, but with reference to a methods publication). No language restrictions were used, and the search period is clearly stated: to January 2008. It bears noting, however, that search terms are not provided or discussed. 

##### iii) Inclusion and exclusion criteria

The inclusion and exclusion criteria are clearly stated, and include a number of specific criteria related to the aim of the study – to review the ethics issues associated with ASCT. First and second screen inclusion criteria include, for example, specification regarding the technology (stem cells), sources (e.g. German National Ethics Council, or others), types of publications (e.g. health economics publications that balance harms and benefits). First and second screen exclusion criteria are likewise provided, and pertain to (among others): publication focus (absence of discussion of ethics issues around ASTC), publication types (e.g. daily newspaper articles), population (e.g. children), and setting (e.g. publications exclusively focused on non-European settings). 

##### iv) Perspective

The review of ethics issues indicates (in the background section) that it is linked to the systematic review of effectiveness of ASTC conducted by IQWiG. Because the analysis utilises the Socratic framework, which includes questions pertaining specifically to various groups of stakeholders (e.g. individual patients, society, physicians, etc.), some information about the perspective can be inferred. The analysis does not appear to take a broad perspective of health service providers, payers, and other members of society. Nor does it appear to take any other specific perspective. The perspective from which the analysis as whole is conducted, is unclear. 

##### v) Ethics framework

The analysis explicitly states that it adopts a modified version of the Socratic approach, consisting of Hofmann’s 33 questions [33]. This choice is justified by the aim of increased transparency and reproducibility, as well as a desire to address each of the 33 questions in more detail. The review, moreover, incorporates a quantitative element, listing how many identified references address each of the 33 questions. Both the choice of the approach and its modifications are explicitly justified. 

#### The Output Domain

##### i) Completeness

The review adopts a quantitative approach – identifying how many publications address each of the 33 questions. This allows for an identification of both the saturation points and the gaps in the literature. Droste et al. found no literature addressing two of the 33 questions – question 8, focusing on whether the technology changes our conception of persons, and question 30, what are the interests of those participating in the technology assessment. Although it is understandable why no literature was found on question 30, the authors of the review do provide an answer about their motivations – these are: a desire to identify harms and benefits, make recommendation for financing this treatment, and methodological development. For question 8, authors acknowledge that this issue may not have been discussed in the literature, i.e. remains a gap. 

##### ii) Bias

The review presents ethics issues in a descriptive manner and the authors do not evaluate, rate, or discuss the issues. However, the study does address several potential biases in the results (that is, the ethics issues identified in the literature) resulting from the applied approach. For example, it identifies some limitations inherent in the adopted approach, such as the potential for the loss of “first-order experiences of patients or patient groups by analysing the (scientific) published literature”. At the same time, they acknowledge that “[t]his is not the case in our example. Our retrieval results included some first-hand reports.” It also notes the bias in favour of the survivors in health-related quality of life data. Moreover, it suggests improvements for future use. 

##### iii) Implications

In adopting the Socratic approach, the study considers a wide range of ethics issues, and their applicability to particular stakeholder groups – individual patients (e.g. challenge to patient autonomy), society (e.g. contesting of social or cultural convictions), care providers (e.g. impact of the technology on the relationship between physicians and patients), third parties (e.g. industry and insurers), etc. However, the policy implications of the analysis as a whole are not identified either generally, or as they apply to the different stakeholder groups. 

##### iv) Conceptual clarification

The systematic review makes no explicit assessment of conceptual issues, and the authors only make some implicit conceptual clarifications in passing. For example, the authors acknowledge the culturally dependence of the understanding of “human dignity”. However, a wide range of ambiguous and vague value-laden concepts – such as autonomy, equality and dignity – are used in the review without clarification. 

##### v) Conflicting values

Because the systematic review utilises the Socratic framework, some of the conflicts of values between various stakeholders are implicitly apparent from the discussions of the individual 33 questions. However, the review does not explicitly reflect on the values implicit in the arguments. Any decision to fund – or not fund – a health technology, is inherently laden with conflicts of values between the stakeholders – patients, healthcare providers, decisions-makers, funders, insurers, industry, and so on. Because the review has as its aim assisting decision-makers in their decision process, this is a shortcoming. 

#### Overall quality assessment

Overall, the elements comprising the Process Domain were completed very well in this review. The research question was clear, and the literature search very comprehensive, although it would have been helpful to have been provided with a list of search terms used, as is common in systematic reviews of clinical effectiveness. The inclusion and exclusion criteria were also very clear. Because impartiality is crucial in ethics analyses, and the perspective from which a technology is assessed will impact both what is included and excluded, and the shape of resultant analysis, the importance of identifying the perspective is considerable. The perspective adopted in the present review is only implicitly suggested, and would benefit from clarification. The ethics framework is clearly identified, and its use to assess completeness is innovative.

The elements of the Output Domain displayed more variability in the degree of completion. Bias issues are considered. On the other hand, policy implications are not clearly drawn out, conceptual clarifications are not offered, and value conflicts are also not clarified. The importance of drawing out policy implications, and value conflicts between the various stakeholders, in ethics analyses conducted for HTA purposes is considerable, as HTA personnel and decision-makers who read such analysis may not be ethicists themselves. The need for conceptual clarification also cannot be underestimated in ethics, which traffics in thick, value-laden concepts. 

## Discussion

To generate the Q-SEA instrument, we adopted a rigorous, three-pronged process, consisting of: a review of guideline documents, an update of a search strategy, and solicitation of the views of experts in methodology in ethics for HTA. As a result, a preliminary version of the Q-SEA instrument has previously been published [10]. However, in applying this instrument to a specific systematic review of ethics issues in HTA, modifications were necessary. In particular, we modified the two domains’ labels, in order to better reflect their foci – internal quality assessment and external quality assessment became process domain and output domain, respectively. Moreover, although we retained many of the elements comprising these domains (e.g. perspective, implications), we modified others (e.g. assumptions, premises, conclusions) as in practice, we found them to be more applicable to evaluating the quality of *individual ethical arguments* than to evaluating *systematic reviews of ethics issues* as a whole. 

Assessment of the quality of systematic reviews in HTA is essential, and ethics analyses conducted for HTA purposes are not exempt from this requirement. We endeavoured to propose an instrument that is usable by both ethicists and non-ethicists alike. However, as the instrument was generated predominantly by individuals with expertise in both ethics and HTA, some elements of the instrument (such as completeness and conceptual definitions) may be more challenging for non-ethicists to assess. The Q-SEA instrument may therefore require further refinements, based on the feedback from its users.

## Conclusion

We propose here Q-SEA, a quality assessment instrument for ethics analyses conducted for HTA purposes, and outline its initial application to an ethics analysis previously conducted for HTA purposes. We believe that the resulting Q-SEA instrument will fill in the thus far neglected methodological gap in ethics for HTA, and that its preliminary application, illustrated above, will assist in its uptake. Further refinements to the instrument are both expected and planned, in light of the experiences of its users in conducting quality assessments of ethics analyses pertaining to various health technologies, conducted for different types of health systems, prepared by HTA agencies with different remits, and so on. The authors therefore welcome the feedback from the instrument’s users.

## Notes

### Competing interests

BH has a potential conflict of interest as author of the Socratic approach that was used as an ethics framework in the Droste et al.’s systematic review, but did not participate in the application of the Socratic approach by Droste et al. Others report no conflicts of interest. 

### Acknowledgements

This paper builds on the discussions held at the “Workshops on Methodology in Ethics for Health Technology Assessments”, which were held in October 2013 in Edmonton and Cologne. The authors gratefully acknowledge the participation and contributions of all of the workshops’ participants. The authors would also like to thank Alric Rüther and Sigrid Droste at the German Institute for Quality and Efficiency in Health Care (IQWiG) and Christa Harstall and Ken Bond at the Institute for Health Economics (IHE) for co-organising and hosting the workshops. 

We would also like to thank the anonymous peer reviewers for their valuable suggestions. 

### Funding

The workshops attracted funding and in-kind contributions from: Alberta Health (Edmonton, Canada); Alberta Innovates-Health Solutions (Edmonton, Canada); Canadian Agency for Drugs and Technologies in Healthcare (Ottawa, Canada); Charles Perkins Centre, University of Sydney (Sydney, Australia); Health Technology Assessment international (HTAi); Institut für Qualität und Wirtschaftlichkeit im Gesundheitswesen (Cologne, Germany); Institute of Health Economics (Edmonton, Canada); International Network of Agencies for Health Technology Assessment (INAHTA); and NHMRC Clinical Trials Centre, University of Sydney (Sydney, Australia).

## Figures and Tables

**Table 1:**
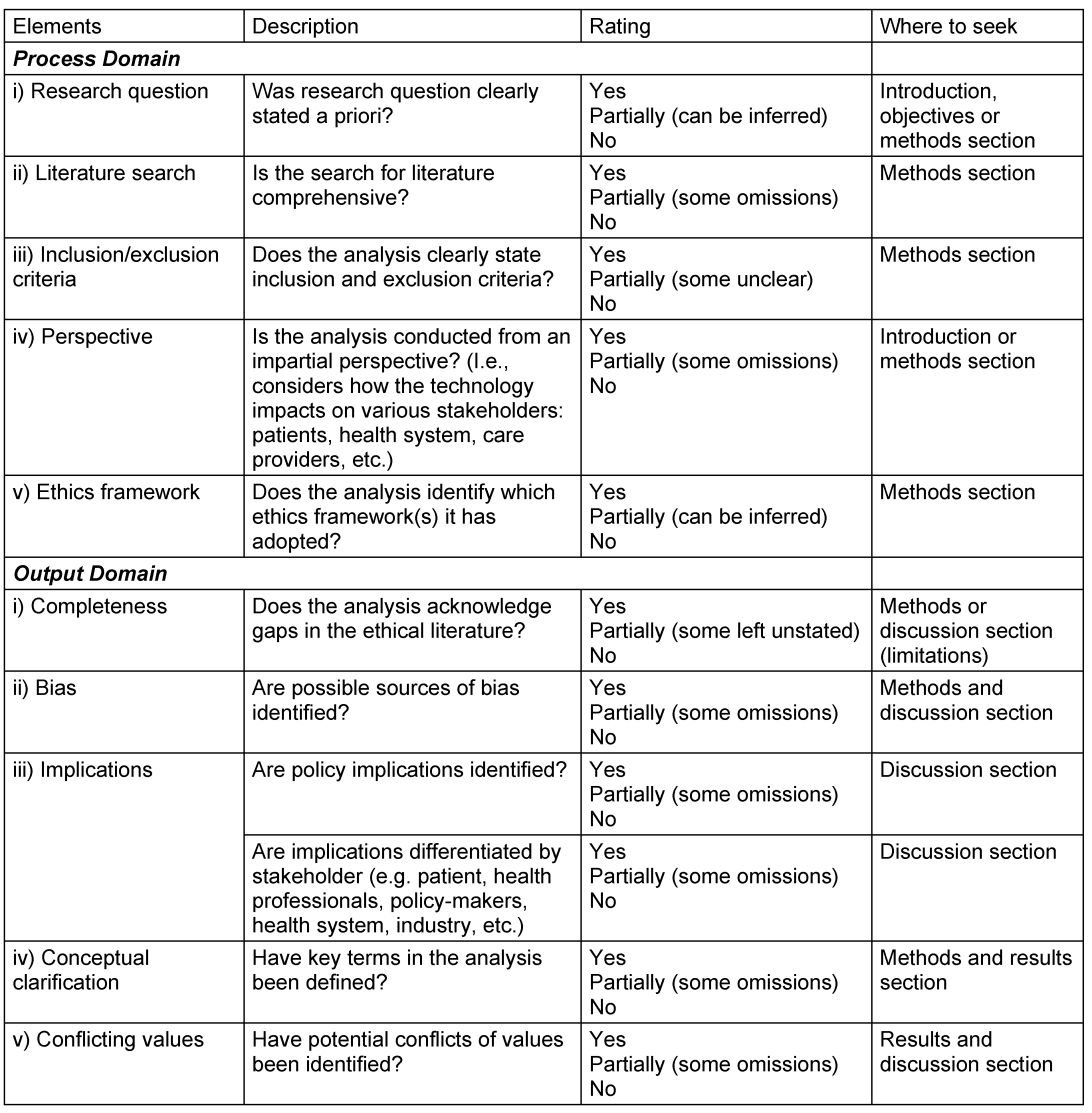
Quality Standards for Ethics Analyses (Q-SEA) in HTA
